# A Case of Recurrent Isolated Uvula Oedema Secondary to Obesity and Obstructive Sleep Apnoea

**DOI:** 10.7759/cureus.29644

**Published:** 2022-09-27

**Authors:** Louis Luke, Rachael Collins, Shyam Gokani, Basil Al-Omari

**Affiliations:** 1 Otolaryngology, James Paget University Hospital, Great Yarmouth, GBR

**Keywords:** isolated uvular angioedema, quincke's edema, obstructive sleep apnoea, obesity, uvula, airway assessment

## Abstract

A 34-year-old male presented as an emergency with sudden onset globus, stertor and choking whilst asleep. He had similar previous episodes that self-resolved. The patient's observations were all within normal range. On examination, he had a grossly enlarged, non-erythematous uvula and there were no signs of respiratory distress or stridor. He was managed with intravenous dexamethasone and an attempt at needle aspiration of the uvula was made but there was no clinical improvement in the patient's condition. Despite no improvement with therapy, he was monitored overnight for any signs of airway compromise and discharged the following morning. His symptoms completely resolved on follow-up in the otolaryngology clinic a week later. He was diagnosed with Quincke’s oedema caused by his obesity and background of obstructive sleep apnoea. We discuss the various aetiologies, assessment, and management of Quincke’s oedema.

## Introduction

Sudden onset isolated angioneurotic uvula oedema, or 'Quincke’s oedema,' is a rare condition with various aetiologies including allergy, medication induced such as non-steroidal anti-inflammatory drugs (NSAIDs) or angiotensin-converting enzyme (ACE) inhibitors, upper respiratory tract infections, uvula trauma, illicit drugs such as cocaine or marijuana, hereditary angioedema, and idiopathic [1]. Patients typically present with a sore throat, odynophagia, dysphagia, gagging and hoarseness but can potentially develop airway obstruction [2]. An important consideration in management is determining the presence of angioedema as this can progress to life-threatening anaphylaxis causing laryngeal oedema. Determining whether the cause is histaminergic or non-histaminergic is important as treatment differs between the two. However, the main priority is securing a safe airway [3].

## Case presentation

**Figure 1:**
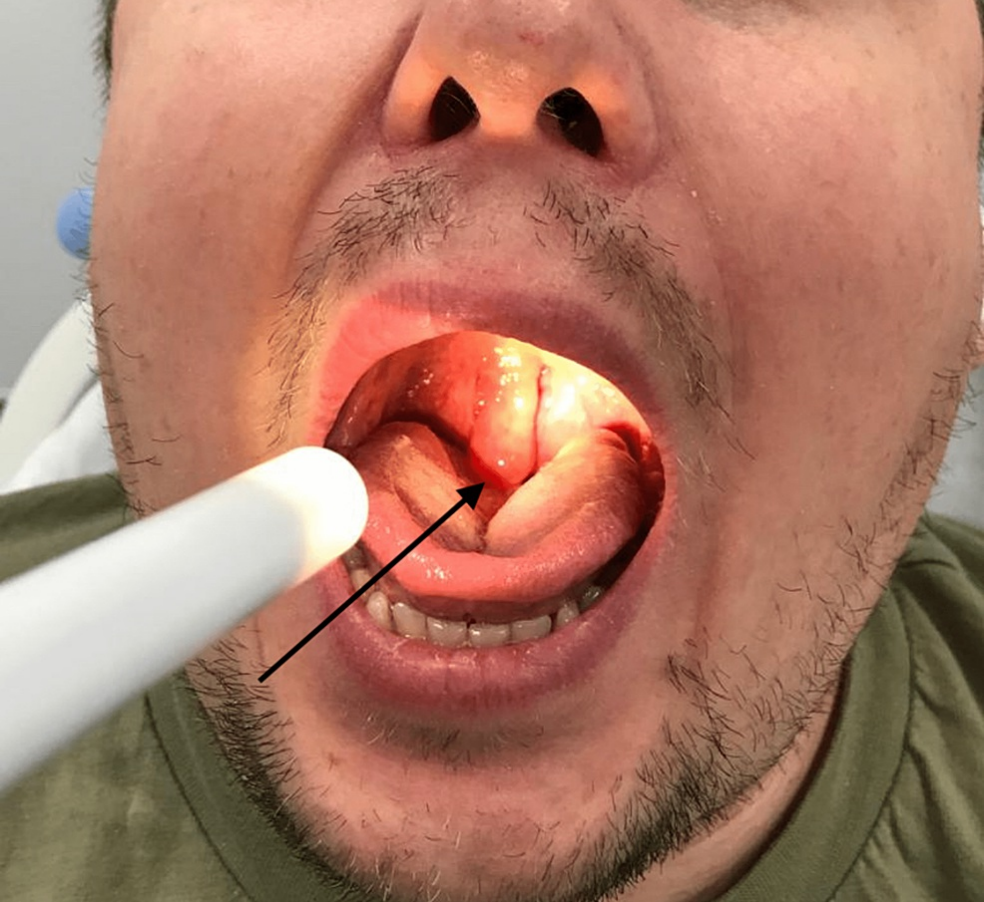
Isolated Uvula Oedema

A 34-year-old male presented in the early hours of the morning to accident and emergency (A&E) with sudden onset foreign body sensation, stertor and choking whilst asleep. From triage, he was transferred to the resuscitation bay. At the time of assessment, he was able to eat and drink and had dysphonia. He denied odynophagia, dyspnoea, fever, otalgia, neck lumps, haemoptysis, night sweats or unintentional weight loss. He reported recent upper respiratory tract infections in his family. He also had three previous self-limiting episodes of a large swollen uvula in the past year and was awaiting an outpatient otolaryngology (ENT) appointment. His past medical history included obesity, mixed anxiety depressive disorder and obstructive sleep apnoea (OSA) diagnosed by polysomnography for which he did not use a continuous positive airway pressure machine. He had no allergies and did not take any regular medications. He worked in an office and was an ex-smoker that stopped five years ago (10 pack years) with minimal alcohol intake. On general examination, his observations were all within normal range and there was no stridor or signs of respiratory distress. Examination of his oropharynx identified a grossly enlarged uvula with no overlying erythema extending to the base of the tongue and touching both palatine tonsils (both tonsils were grade one in Brodsky's tonsillar grading scale) as can be seen in Figure 1. His neck was soft and non-tender with no cervical lymphadenopathy. Flexible nasendoscopy at the bedside revealed an isolated non-erythematous and swollen uvula. Blood tests showed an elevated C-reactive protein (52) with other blood test results being unremarkable. The patient was managed with 8 mg intravenous (IV) dexamethasone but did not improve. Needle aspiration under local anaesthetic was attempted, but no fluid was drained. Following this, he was given another dose of 8 mg intravenous dexamethasone but there was no improvement in the appearance of the uvula. As he remained well overnight, he was discharged home with follow-up in the ENT clinic and safety-netting advice. 

On review in the ENT outpatient clinic a week later, he reported spontaneous resolution of his symptoms within one day of discharge. There were no identifiable triggers such as inhaled or ingested allergens from the history and on examination his uvula was normal. A clinical diagnosis of Quincke’s oedema secondary to obesity and OSA was made from the patient’s history and presentation. No further management was required, and the patient was discharged back to the care of his general practitioner. 

## Discussion

Quincke’s oedema is typically characterised by rapidly developing, localised, recurrent uvula angioedema causing symptoms such as globus, difficulty talking and swallowing that resolves in hours and days. It is usually caused by a type 1 hypersensitivity reaction [2]. The top priority in management is to ensure the airway is safe. Taking a focused history asking questions about personal or family history of atopy or angioedema and performing investigations including full blood count, urea and electrolytes, liver function tests, thyroid function tests, serum tryptase and immunoglobulin E (IgE) can help to identify triggers [1]. 

Determining whether there is concomitant angioedema is important due to the risk of progression causing life-threatening airway compromise. Two classifications of angioedema exist with different treatments. In the majority, histaminergic angioedema with mast cell degranulation occurs, and histamine and inflammatory mediators are released This is usually a type I IgE-mediated hypersensitivity immune response. Urticaria usually occurs and there is the potential of developing life-threatening anaphylaxis [4]. Management with antihistamines, corticosteroids and adrenaline is effective. Conversely, non-histaminergic angioedema presents without urticaria and is bradykinin and complement mediated. ACE inhibitors block the normal degradation of bradykinin whilst hereditary angioedema patients generally have C1-inhibitor deficiency resulting in the accumulation of bradykinin [3]. Considering this, treatment with bradykinin receptor antagonists or complement C1-inhibitor concentrate should be used instead [5]. If suspected, screening for C1-inhibitor deficiency can be performed as part of the assessment looking for normal C3 levels and low levels of C4 [2]. When the presentation is acute and severe with potential airway compromise, prompt assessment and airway management including intubation or possible emergency front of neck access such as cricothyroidotomy or tracheostomy may be indicated [5]. Very rarely, surgery is used to manage recurrent or refractory cases. Examples in the literature include, using a needle to make several lacerations to the uvula or a partial uvulectomy [6,7]. However, there is a paucity of evidence of using invasive techniques to manage uvula oedema. 

Besides angioedema and allergy-related causes of uvula oedema, infective causes such as uvulitis and epiglottitis need to be considered in the differential when a patient presents with uvula cellulitis and fever. Therefore, directly visualising the larynx to rule out epiglottitis and ensuring the airway is patent is necessary in addition to antibiotic therapy to treat bacterial causes [8].

In the literature, over half of the cases of Quincke’s oedema were found to be idiopathic. Interestingly, high body mass index and snoring were predisposing factors for these recurring episodes [1]. Furthermore, there are cases of snoring-induced uvula oedema in conjunction with other causes such as the concurrent use of ACE inhibitors, deep sedation or in hereditary angioedema [5,9,10]. Snoring is a key sign of partial airway obstruction in obstructive sleep apnoea (OSA). Uvula inflammation characterised by plasma cell infiltration and interstitial oedema has been shown to be present in patients with moderate OSA which may play a role in patients presenting with recurrent uvula oedema [11]. 

In this case, since there were no obvious triggers such as a history of allergy, medication use or infection, the likely cause of this uvula oedema was secondary to snoring, supported by the fact the patient’s onset of symptoms occurred whilst he was asleep. There are several learning points from the assessment and management of this case. Firstly, appropriate treatment with IV dexamethasone was given to manage potential upper airway obstruction in a safe environment, the A&E resuscitation bay. However, conducting further blood investigations for angioedema to determine a likely causative factor was not performed. Furthermore, there is limited evidence for the need for needle aspiration as this has only been useful in draining a peritonsillar abscess with associated uvula hydrops [12]. As this patient did not improve with IV dexamethasone alone, it might have been worth considering using complement C1-inhibitor concentrate for non-histaminergic angioedema as there was no associated urticaria or history of allergy. In the outpatient setting, consideration for a referral to respiratory and allergy physicians may have been useful in helping control the patient’s OSA and to conduct further investigations to rule out other causes.

## Conclusions

Quincke’s oedema or isolated uvula oedema has a range of causes of which the majority are idiopathic. From this case and in the literature, we have shown that snoring, being overweight, and having OSA can predispose patients to recurrent episodes. It is important to be aware of infectious, histaminergic, and non-histaminergic angioedema causes of uvula oedema as their treatments vary. Overall, a thorough clinical assessment, prompt airway management and appropriate investigations can help identify a causative factor and manage patients with a good prognosis.
